# Structural Characterization and Study of the Mixed-Ion Effect in K–Li Metaphosphate Glasses

**DOI:** 10.1021/acsomega.5c01027

**Published:** 2025-04-11

**Authors:** Izabel Mateus Nogueira dos Santos, Flavio Augusto de Melo Marques, Adriana Marcela Nieto Munõz, Ana Candida Martins Rodrigues, José Fabian Schneider, Jefferson Esquina Tsuchida

**Affiliations:** † Universidade Federal de Lavras (UFLA), Departamento de Física (DFI), Campus universitário UFLA, Lavras, Minas Gerais 37200-000, Brazil; ‡ Universidade Federal de São Carlos, Departamento de Engenharia de Materiais, Rod Washington Luis Km 235, São Carlos, São Paulo, BR 13565-905, Brazil; § Universidade de São Paulo, Instituto de Física de São Carlos, Física e InformáticaAv. Trabalhador Saocarlenses, São Carlos, São Paulo, BR 13566-590, Brazil

## Abstract

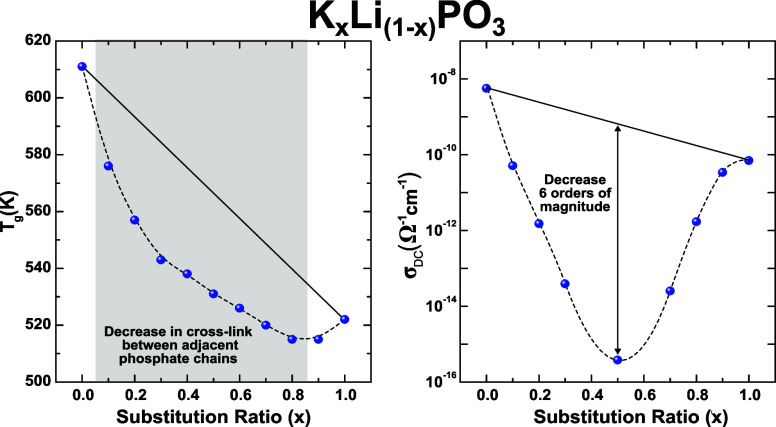

This study investigates potassium–lithium metaphosphate glasses using Differential Scanning Calorimetry, Complex Impedance Spectroscopy, Nuclear Magnetic Resonance, and Raman Spectroscopy to elucidate the structural mechanisms underlying the Mixed Ion Effect. Thermal analyses reveal a systematic decrease in the glass transition temperature with increasing potassium content, which is dictated not solely by ionic size mismatch but also by structural reorganization within the glass network. The redistribution of nonbridging oxygens and the reduction of phosphate cross-links contribute to this behavior. Impedance spectroscopy shows a pronounced nonlinear reduction in ionic conductivity, decreasing by over 6 orders of magnitude at room temperature for the intermediate composition. NMR analysis indicates a nearly linear evolution of ^31^*P* and ^7^*Li* chemical shifts and full width at half-maximum, confirming the absence of phase segregation. Raman spectroscopy reveals a consistent *PO*_2_ symmetric mode shift, indicative of solid solution behavior and random cation mixing. These findings validate two key hypotheses of the Random Ion Distribution Model: the structural specificity of each cation site and their random distribution within the glass network, while also demonstrating that structural reorganization plays a critical role in modulating *T*_g_.

## Introduction

Phosphate glasses have drawn the attention of researchers owing to their exceptional properties, including low melting point, high ionic conductivity, good optical transparency, high expansion coefficient, and the ability to dissolve substantial quantities of alkali metal oxides. As a result, these glasses have significant potential for various applications in optics, electronics, and energy storage. Their distinctive features make them promising materials for several technological products, such as solid-state laser matrices,^1,2^ industrial waste immobilizers,^3,4^ low-temperature seals,^5,6^ and others. Therefore, the investigation of the structure and optical and electrical properties of these vitreous systems is important for the development in this field, since these properties are intrinsically related to many other properties, such as density, mechanical resistance, chemical stability, and thermal conductivity, among others.

The mixture of some species of metal ions in a glass matrix can originate a nonlinear behavior in some properties of the glass. These nonlinearities can present minimum and maximum depending on the concentration of each metal ion mixed in the matrix; this effect is called the Mixed Ion Effect (MIE).^7,8^ Several pairs of metal ions have already been observed to cause the MIE.^9−12^

Most of the models devised to explain the origin of the MIE assume that the mobile ions are randomly distributed in the glass matrix. The Random Ion Distribution Model (RIDM) is a model that assumes ions maintain their local sites fixed in relation to the glass matrix, leading to a differentiation among different ionic sites and a significant reduction in the number of sites available for ionic migration.^13−18^ When alkaline ions are randomly mixed, this distribution within the glass network can block conduction pathways due to the nonidentical local structural environments and the resulting energy mismatch for ionic migration. This energy mismatch makes it less likely for ions to make energetically favorable jumps, increasing the activation energy and lowering the ionic conductivity. Furthermore, the random distribution of ions can cause partial obstruction of diffusion pathways, as ions become entrapped and are compelled to diffuse through routes with higher energy barriers compared with single-alkali glasses.

According to the RIDM theory, the reduction in ionic conductivity is caused by the difference in ion sizes, where the varying dimensions of the local sites in the vitreous matrix inhibit ion migration, leading to the MIE depending on the local structure of the glass and the random mixture of ions. Although the RIDM provides a well-established explanation for the MIE in ionic conductivity, previous studies have shown that ion size mismatch alone does not fully account for the intensity of the MIE in properties such as *T*_g_.^19^ This suggests that additional factors may influence the behavior of *T*_g_ in mixed-ion glass systems.

The objective of this study is to investigate, using Raman spectroscopy and NMR techniques, the relationship between the cation distribution and the structural properties that are crucial to the development of the MIE. This includes analyzing the structural specificity of the sites occupied by each cation species and their random distribution within the glass network. The glass system analyzed in this study is the mixed metaphosphate *K*_*x*_*Li*_(1–*x*)_*PO*_3_, with 0 ≤ *x* ≤ 1, where the monovalent cation pair K and Li exhibit a significant size mismatch. In the current analysis, ^31^*P* and ^7^*Li* solid-state NMR techniques are used to obtain information about the local environment around ^31^*P* and ^7^*Li*, as well as the distribution of Li in the glass network. Raman spectroscopy was employed as an important tool to evaluate the random distribution of K and Li in the glass network.

## Experimental Section

Glasses with the composition *xKPO*_3_·(1–*x*)*LiPO*_3_, with 0 ≤ *x* ≤ 1, were prepared by mixing crystalline metaphosphate powders in the desired ratios. Crystalline metaphosphates were synthesized using the methodologies described as follows. Potassium metaphosphate (*KPO*_3_) was obtained by heating *K*_2_*HPO*_4_ powder (Synth, 99%) at 200 °C for 2 h and sequentially for 3 h at 750 °C in porcelain evaporating dishes. Lithium metaphosphate (*LiPO*_3_) was prepared by mixing lithium carbonate (*Li*_2_*CO*_3_, Synth, 99%) with *NH*_4_*H*_2_*PO*_4_ (Neon, 98%) in porcelain evaporating dishes and slowly heating to 200, 400, and 600 °C, with 2 h stabilization periods at each one of these three temperatures.

The resulting polycrystalline samples were checked with powder X-ray diffraction (XRPD), using Cu Kα radiation and a scanning 2θ range from 10° to 90° with 0.02° resolution (Shimadzu XRD-6100). The desired crystalline phases were corroborated with XRPD analysis, referencing Crystallography Open Database (COD) entries 2107006 for *KPO*_3_ and 2107072 for *LiPO*_3_.

The glasses were prepared in an adequate proportion of metaphosphate powders in porcelain crucibles at 950 °C for 20 min. Transparent glass pieces were obtained by pouring the melt on a steel block at room temperature without any annealing. The samples were stored in desiccators.

Differential Scanning Calorimetry (DSC) has been used to determine the glass transition temperature (*T*_g_), using a NETZSCH DSC404 calorimeter with the temperature varying from room temperature to 1000 °C by applying a heating rate of 10 °C/min under an uncontrolled atmosphere in platinum crucibles.

Electrical properties were acquired by using impedance spectroscopy with a Solartron 1260 Impedance/Gain Phase Analyzer coupled with a Solartron 1296 Dielectric Interface, which, depending on the frequency, allows the measurement of impedances of up to 1 × 10^8^ Ohms. Measurements were obtained by using a two-electrode configuration in an air atmosphere with a frequency range between 10^6^ and 10^–1^ Hz. The temperature was controlled from room temperature to 200 °C with an accuracy of ±0.1 °C. For electrical characterization, samples were coated with gold thin-film electrodes on both parallel surfaces using a sputtering method.

Raman scattering measurements were performed on a LabRAM HR Evolution micro-Raman spectrometer from Horiba Scientific using a 532 nm line of the Nd:YAG laser in the backscattering geometry at room temperature in the region from 10 to 1700 cm^–1^, with five accumulations and an acquisition time of 20 s.

High-resolution Nuclear Magnetic Resonance (NMR) spectroscopy was carried out at a magnetic field of 9.4 T on a Varian Unity Inova spectrometer using a 4 mm probe with Magic Angle Spinning (MAS) up to 10 kHz with the samples packed inside silicon nitride rotors. For ^31^*P*-NMR, π/2 pulses of 3 μs and recycle delays of up to 120 s were used, whereas for ^7^*Li*-NMR, π/2 pulses of 2.5 μs and recycle delays of up to 50 s were used. Chemical shift references for ^31^*P* and ^7^*Li* were aqueous solutions of 85% *H*_3_*PO*_4_ and 1 M LiCl, respectively.

## Results

### Differential Scanning Calorimetry (DSC)

**Figure 1:**
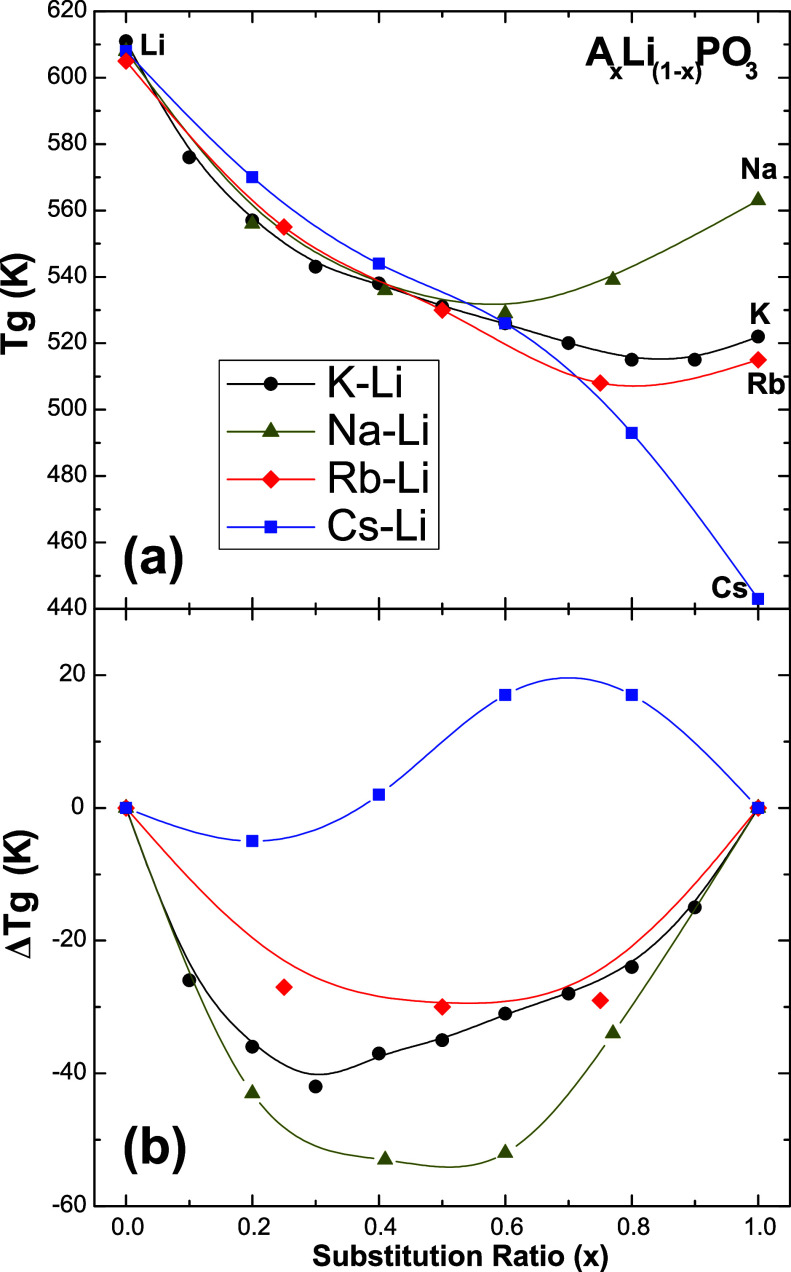
(a) Glass transition temperature (*T*_g_) as a function of composition for the K–Li system compared to data from other mixed-alkali metaphosphate glasses (Li–Na, Rb–Li, and Cs–Li). (b) Glass transition temperatures plotted as differences from the linear interpolation between the two corresponding single-alkali glasses. Adapted with permission from Refs^11,19^. Copyright 2017 and 2013-2014 Royal Society of Chemistry. Full lines are guides for the eyes.

Figure 1a shows the measured values of the glass transition temperatures *T*_g_ for the K–Li metaphosphate glasses. To facilitate the discussion and for comparison purposes, we have provided the *T*_g_ data of the previously reported glass series: Na–Li,^9^ Rb–Li,^11^ and Cs–Li^19^ are also included. The values of *T*_g_ for these glass systems are plotted as a function of the cation substitution ratio *x* = [*A*]/[*Li* + *A*], where *A* represents Na, K, Rb, or Cs. Considering just the single-alkali glasses, the reduction in *T*_g_ values observed in the sequence *LiPO*_3_ → *NaPO*_3_ → *KPO*_3_ → *RbPO*_3_ → *CsPO*_3_ is associated with the increment of the ionic radius (0.74, 1.02, 1.38, 1.49, and 1.70 Å, respectively).^20^ This behavior can be attributed to the decreased Coulombic interaction strength between the cations and the nonbridging oxygens (NBOs). Cations with higher ionic potential tend to form cross-links between different metaphosphate chains. Therefore, cations with larger ionic radii have weaker potentials and form fewer cross-links, resulting in increased mobility of the metaphosphate chains and consequently a lower *T*_g_. This reduction in interaction strength is similarly noted in mixed glasses as smaller cations are replaced by larger ones. The nonadditive behavior observed in *T*_g_ reveals the presence of MIE. This effect is clearly evidenced in Figure 1b, where the glass transition temperature (*T*_g_) was represented as the difference (Δ*T*_g_) between the values of linear interpolation and the values in the single-alkali glasses. These results indicate a consistent weakening of the glass structure in mixed systems. This phenomenon has been attributed to the dynamic coupling between mobile ions, facilitated by the glass matrix.^21^

### Impedance Spectroscopy

**Figure 2:**
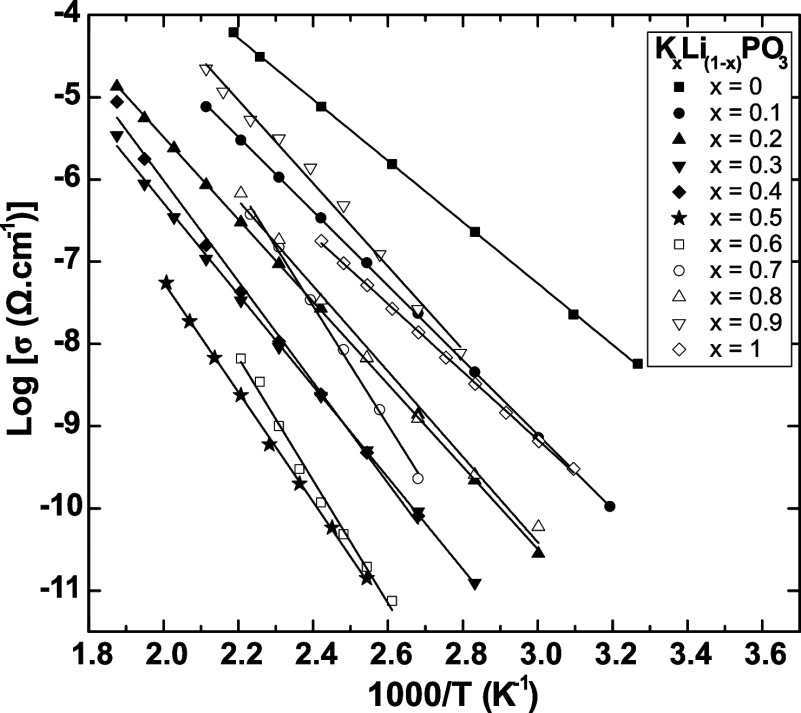
Arrhenius plot of log(σ) for the *K*_*x*_*Li*_(1–*x*)_*PO*_3_ glasses. Full lines represent fitting using eq 1.

Impedance is a complex number that may be represented by a Nyquist plot. The ionic conductivity, σ, of all samples could be obtained by exploring the complex plot in Cartesian coordinates of the impedance, as described elsewhere.^11^ Exploring a range of temperatures allows us to represent the log of ionic conductivity as a function of the inverse temperature, the so-called Arrhenius plot, as shown in Figure 2. The linear decay of log(σ) as a function of the inverse temperature indicates Arrhenius behavior below the glass transition temperature. The data were fitted using the following linear equation:
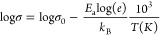
1where *k*_B_ is the Boltzmann constant, σ_0_ is the pre-exponential factor of the Arrhenius expression, and *E*_a_ is the activation energy for conduction.

**Figure 3:**
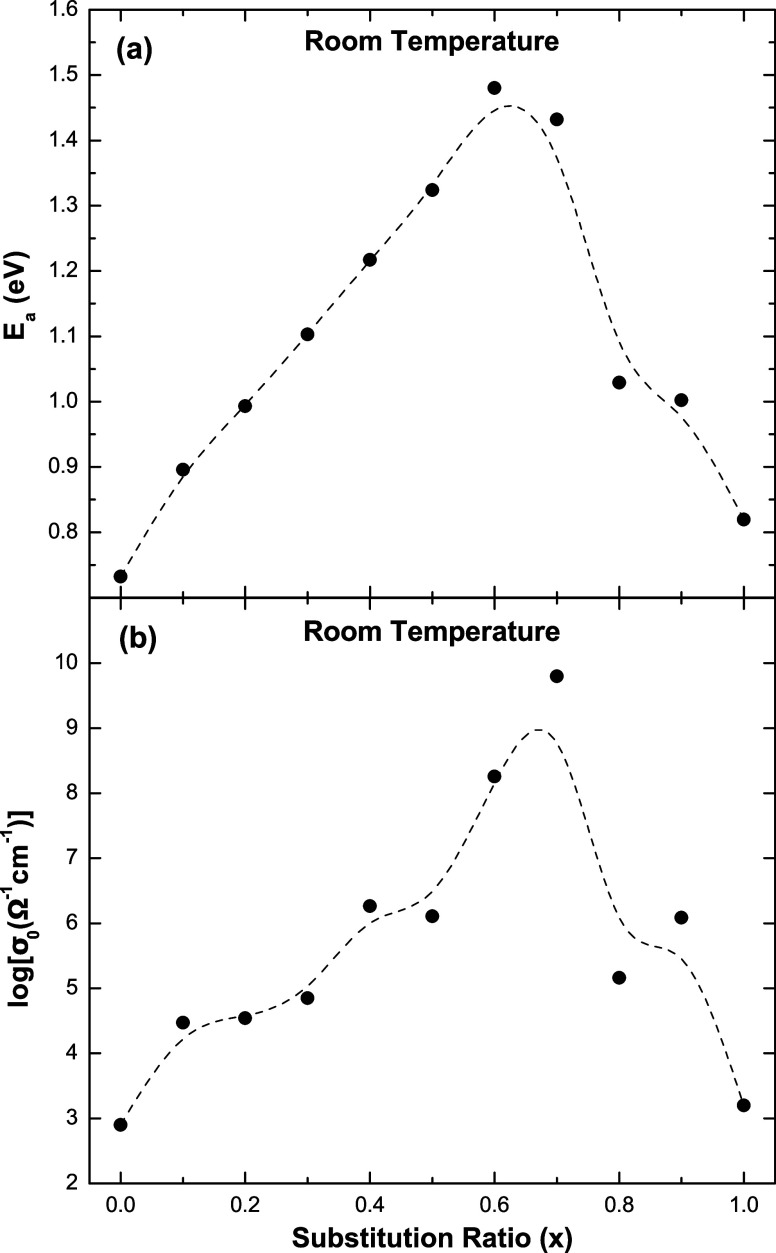
(a) Activation energy and (b) pre-exponential factor obtained from linear fitting of the Arrhenius plot in Figure 2. Dashed lines are guides to the eyes.

Figure 3a,b shows the activation energies and the pre-exponential factors, respectively, obtained from the linear fit of the Arrhenius plot (Figure 2) as a function of the substitution ratio of *K* and *Li* ions (*x* = [*K*]/[*K* + *Li*]). The activation energy increases with a higher substitution ratio, reaches a maximum at the composition *x* = 0.6, and then decreases. Similar behavior was previously observed in K–Na metaphosphate glasses.^11^ The pre-exponential factor exhibits similar behavior of *E*_a_ with a maximum at the substitution ratio *x* = 0.7.

**Figure 4:**
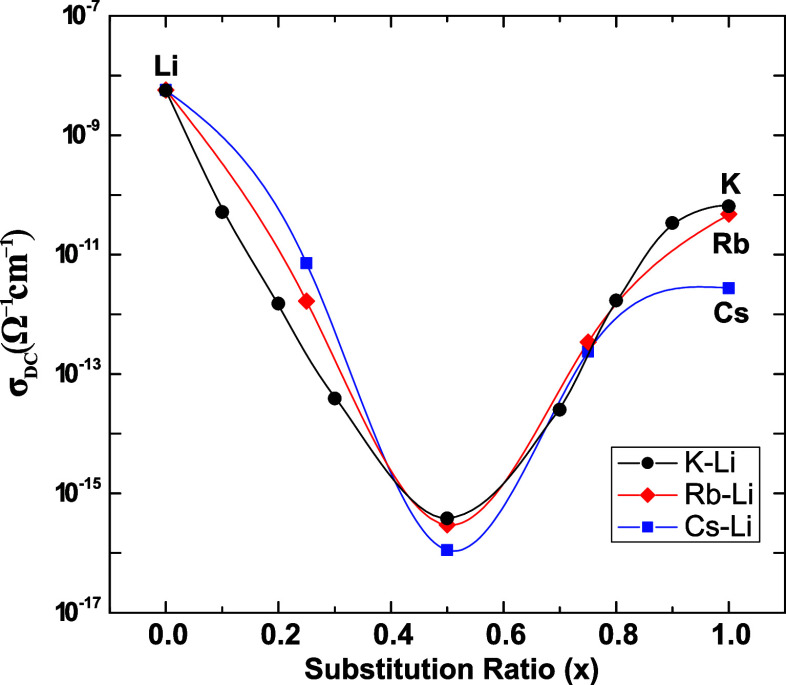
Ionic conductivity at room temperature as a function of the substitution ratio for the *K*_*x*_*Li*_(1–*x*)_*PO*_3_ glasses compared to data from Rb–Li and Cs–Li mixed-alkali metaphosphate glasses. Adapted with permission from Refs^11,19^. Copyright 2017 and 2013 Royal Society of Chemistry. Full lines are guides to the eyes.

Figure 4 shows the ionic conductivity as a function of the substitution ratio at room temperature (33 °C). To facilitate the discussion and for comparison purposes, the σ_DC_ data of the previously reported glass series, Rb–Li and Cs–Li,^11^ are also included. Values of σ_DC_ show a strong deviation with respect to additive behavior, reaching a minimum (about 10^–15^ Ω^–1^ cm^–1^) at the composition *x* = 0.5, and then increase up to 10^–10^ Ω^–1^ cm^–1^. This deviation of σ_DC_ from additivity, of 6 orders of magnitude, is one of the strongest MIEs observed in glasses.

This behavior is consistent with previously reported studies^11^ and is due to the fact that diffusion pathways are constituted by sites specifically adapted to accommodate each ion species. Therefore, the random mixture of *Li* and *K* in the glass network causes the diffusion pathways of *Li* to be obstructed by the presence of the diffusion pathways of *K*, and the degree of obstruction is higher in the intermediate composition with *x* = 0.5.

**Figure 5:**
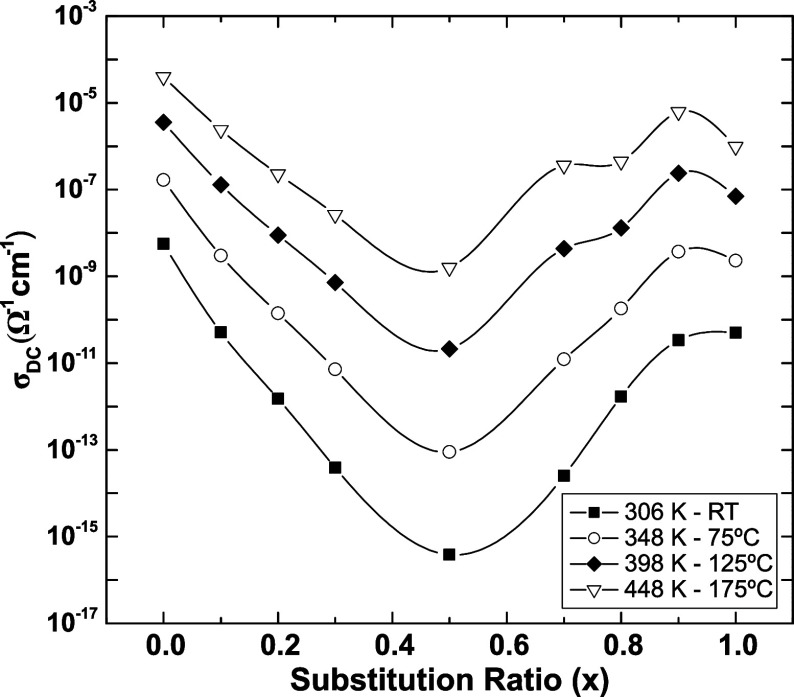
Ionic conductivity at different temperatures as a function of the substitution ratio for the *K*_*x*_*Li*_(1–*x*)_*PO*_3_ glasses. Full lines are guides to the eyes.

Ionic conductivity is commonly understood to increase at higher temperatures owing to the rise in the thermal energy of ions, which raises the probability of hopping. This dependence makes MIE strongly affected by temperature. Figure 5 shows a decrease in MIE magnitude as the temperature increases from room temperature up to 175 °C. The ionic conductivity shows a variation of 6 orders of magnitude at room temperature, which decreases down to 4 orders of magnitude. It should be noted that the ionic conductivity has an exponentially increasing dependence on the activation energy, as shown in eq 1. Thus, a higher activation energy causes the ionic conductivity to increase much faster with increasing temperature, allowing for greater ion mobility and resulting in a decrease in the intensity of the MIE with increasing temperature.

### Raman Spectroscopy

**Figure 6:**
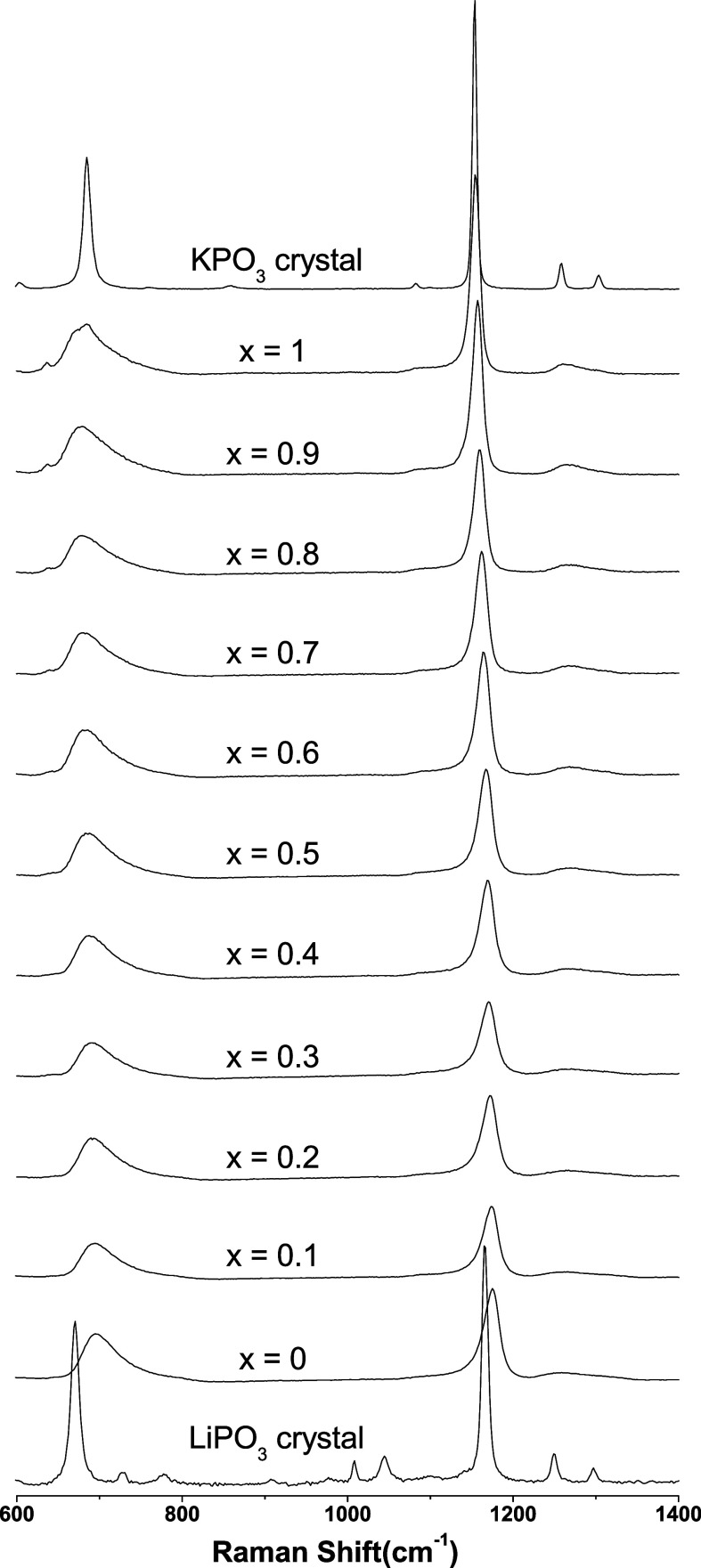
Raman spectra for the *K*_*x*_*Li*_(1–*x*)_*PO*_3_ glasses and the respective crystalline compounds of the simple alkaline glasses.

Figure 6 shows the Raman spectra for the K–Li metaphosphate glasses and the *LiPO*_3_ and *KPO*_3_ crystalline compounds. The region between 600 and 1400 cm^–1^, as shown in Figure 6, presents peaks related to alkali metaphosphate crystals and glasses as described elsewhere in the literature.^22−25^ Two peaks are observed in the Raman spectra of the alkali metaphosphate glasses: one peak between 600 and 700 cm^–1^ and another intense peak in the range of 1000–1200 cm^–1^, which are assigned to ν(*P*–*O*–*P*) stretching vibrations from the bridging oxygen (BO) of *Q*^2^ phosphates and ν(*PO*_2_) stretching vibrations involving the NBO from *Q*^2^ phosphates, respectively. For high wavenumbers, between 1200 and 1300 cm^–1^, there is also a less intense narrow peak at approximately 1250–1270 cm^–1^ that is attributed to asymmetric ν(*PO*_2_) modes. The frequency region between 1050 and 1130 cm^–1^ corresponds to ν(*PO*_3_) stretching vibrations from end-group *Q*^1^ units.

**Figure 7:**
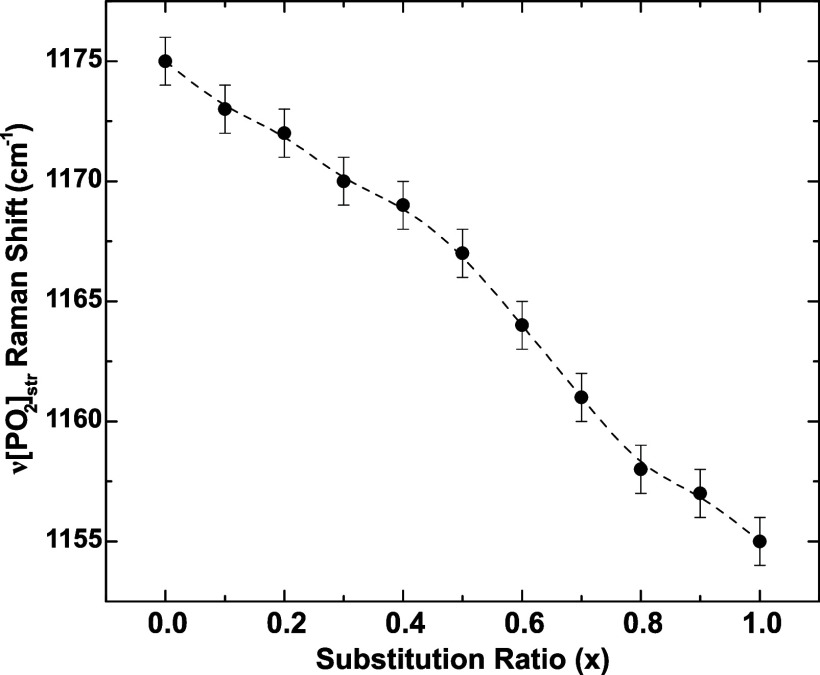
Raman shift of the ν(*PO*_2_) symmetrical vibrational mode as a function of the substitution ratio for the *K*_*x*_*Li*_(1–*x*)_*PO*_3_ glasses. Dashed line is a guide to the eyes.

Figure 7 shows the evolution of the Raman shift for the (*PO*_2_) symmetric mode as a function of the substitution ratio. The Raman shift, for 0< *x* < 1, assumes intermediate monotonic values between the frequencies of compositions *x* = 0 and *x* = 1, which is typical of the behavior of solid solutions. This behavior indicates random site occupation, providing evidence for ionic mixing within the glass network, which is one of the fundamental structural hypotheses used in models to explain MIE in conductivity. The Raman shift of the symmetrical vibrational mode ν(*PO*_2_) decays slightly and linearly with increasing substitution ratio, which is correctly associated with the differences of the *Li*^+^ and *K*^+^ potentials. The vibrational frequency varies depending on the cation species bonded to the terminal oxygen, taking higher values for cations with a higher potential. The increased covalency of the cation–oxygen bonds explains this effect.^26^ The ν(*PO*_2_) vibration frequency decreases sharply when *K*^+^ replaces *Li*^+^, due to the decrease in cationic potential.

### ^31^P-MAS-NMR

**Figure 8:**
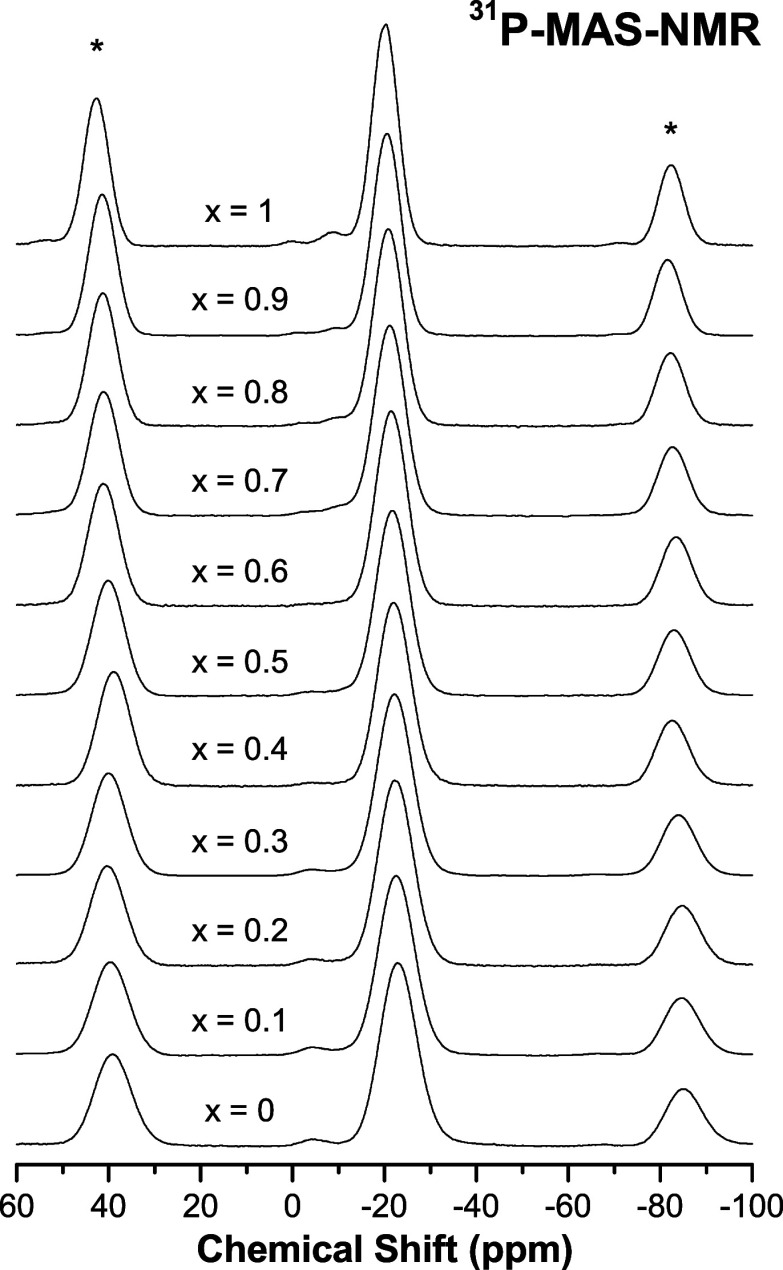
^31^*P*-*MAS*-*NMR* spectra for the *K*_*x*_*Li*_(1–*x*)_*PO*_3_ glasses.

**Figure 9:**
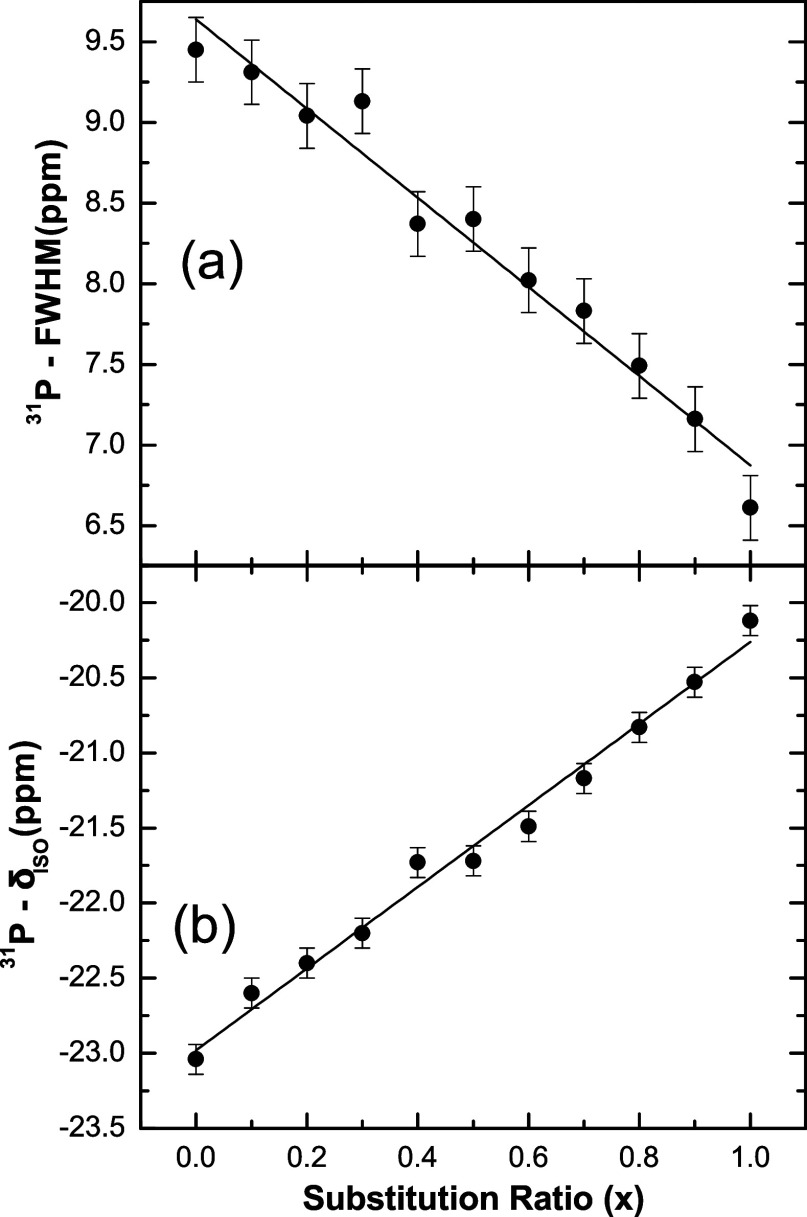
^31^*P*-*MAS*-*NMR* parameters measured for the *Q*^2^ resonance in *K*_*x*_*Li*_(1–*x*)_*PO*_3_ glasses: (a) FWHM and (b) isotropic chemical shift (δ_ISO_). The lines are linear fittings of the respective data.

Figure 8 displays the ^31^*P*-*MAS*-*NMR* spectra for K–Li metaphosphate glasses with various substitution ratios. It is evident from the results that there is a dominant peak that corresponds to *Q*^2^ tetrahedra and a minor peak with a higher chemical shift, which represents the *Q*^1^ tetrahedra. The presence of a fraction of *Q*^1^ species is due to slight deviations from stoichiometry, caused by the presence of *OH* groups in the starting materials or hydrolysis that occurred during the experiments, which is typical for metaphosphate compositions. Figure 9a,b depicts the Full Width at Half-Maximum (FWHM) and the central isotropic chemical shift (δ_ISO_) of the ^31^*P*-*MAS*-*NMR* line, respectively. The inhomogeneous line broadening of the ^31^*P* resonance is caused by the distribution of isotropic chemical shifts due to structural disorder. A linear increase in shielding of around 3 ppm is observed upon comparison of the pure *K* and *Li* metaphosphate glasses. The FWHM displays a slight nonlinear behavior, with the *K* content decreasing up to the *KPO*_3_ glass and resulting in a narrowing of around 3 ppm throughout the entire compositional range. The smaller ^31^*P* linewidth in the *KPO*_3_ glass compared to the *LiPO*_3_ glass, observed here, is consistent with previous research on metaphosphate glasses,^11,27^ as small cations with high ionic potentials such as *Li*^+^ interact more strongly with NBO, leading to a wider spread of local environments than larger cations such as *K*^+^. This trend is reflected in the values of δ_ISO_ shown in Figure 9b, where the linear increase as a function of the exchange of *Li*^+^ ions for *K*^+^ is due to the fact that ions with lower cationic potential systematically decrease the shielding of ^31^*P*, owing to the lower intensity of O–Me bonds.

### ^7^Li-MAS-NMR

**Figure 10:**
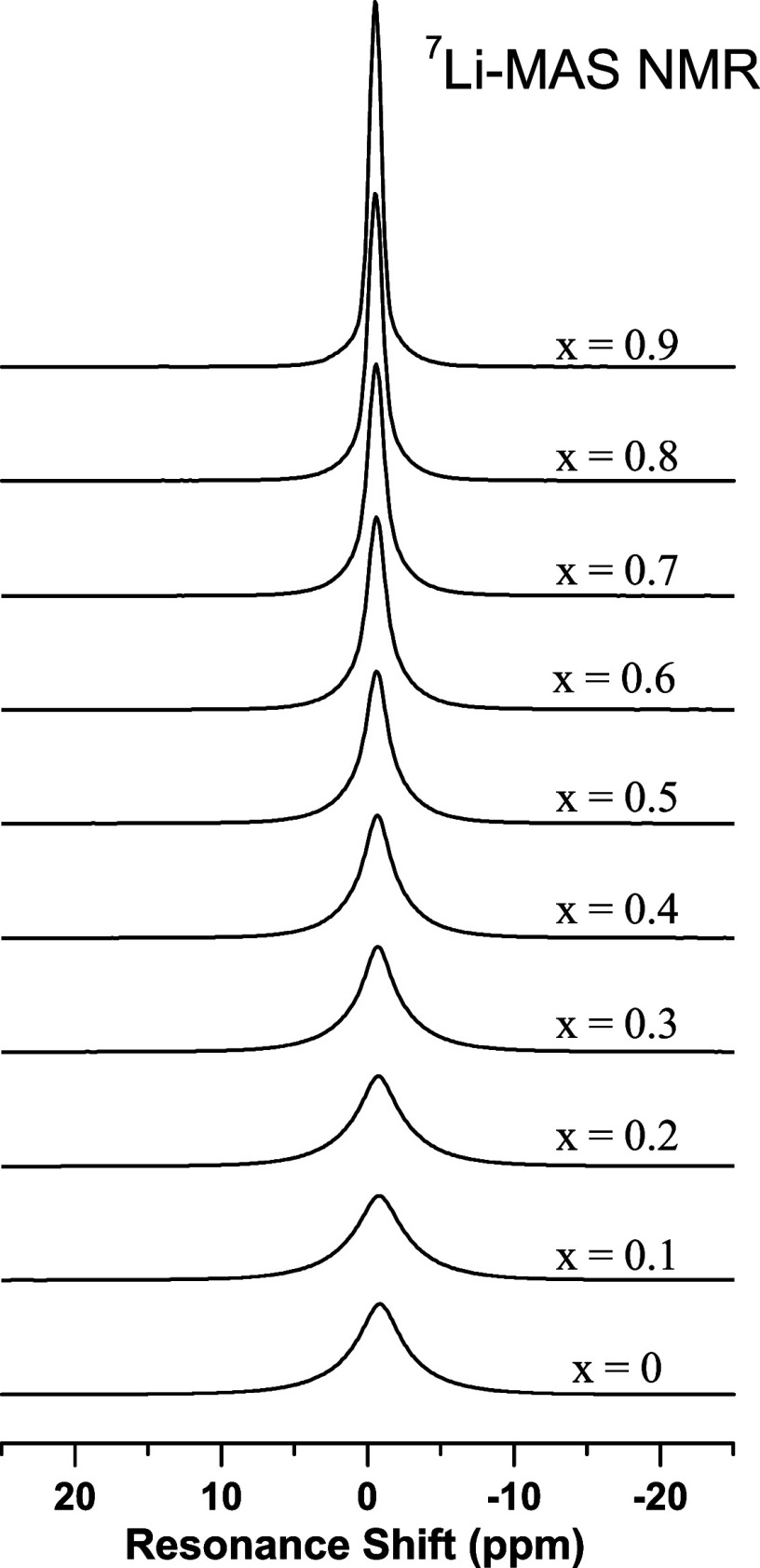
^7^*Li*-*MAS*-*NMR* spectra for the *K*_*x*_*Li*_(1–*x*)_*PO*_3_ glasses.

**Figure 11:**
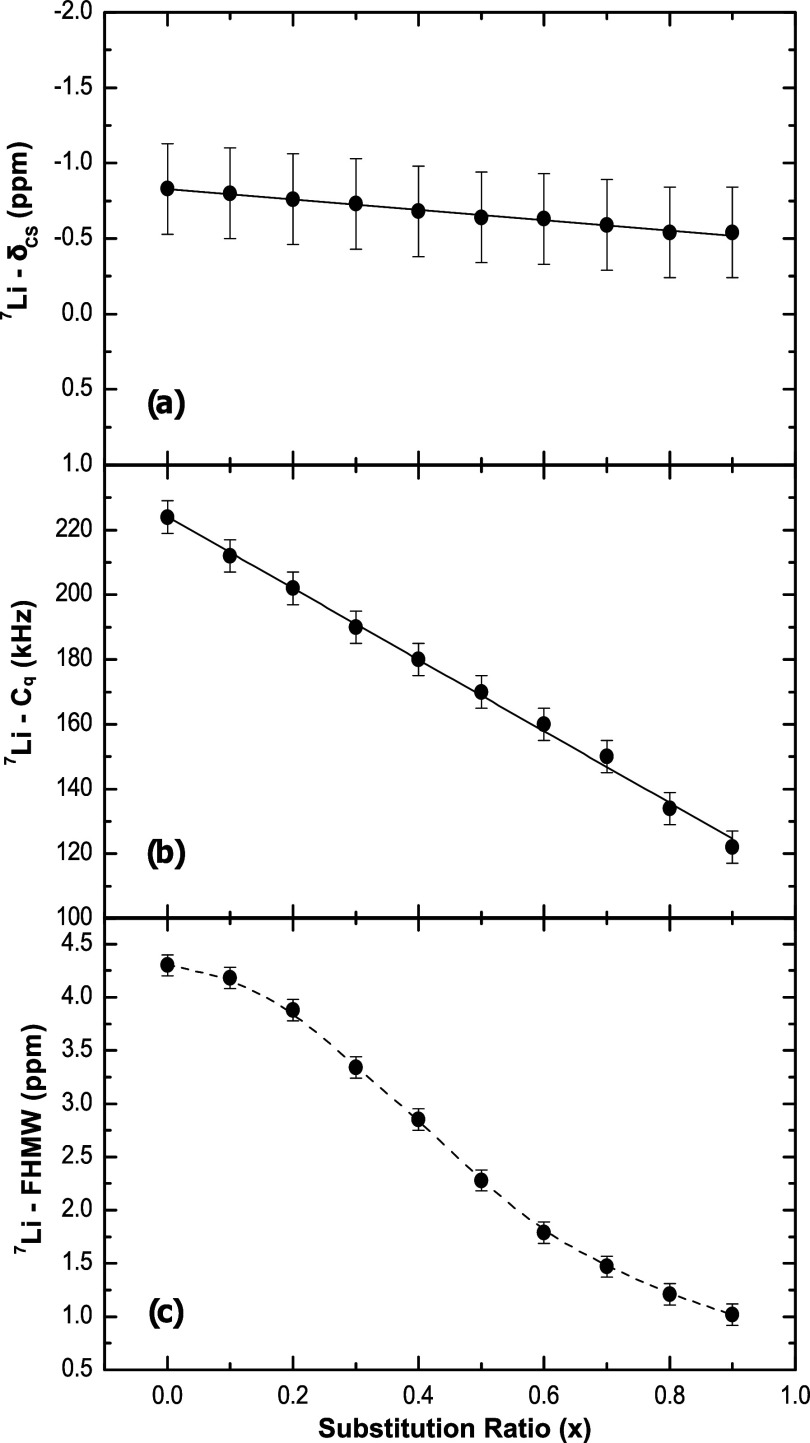
^7^*Li*-*MAS*-*NMR* parameters measured for *K*_*x*_*Li*_(1–*x*)_*PO*_3_ glasses: (a) average isotropic chemical shift (δ_CS_), (b) average quadrupolar coupling constant (*C*_q_), and (c) full width at half-maximum (FWHM). The straight line is a linear fitting of the data, and the dashed line is a guide to the eyes.

Figure 10 shows the ^7^*Li*-*MAS*-*NMR* spectra for the series of *K–Li* metaphosphate glasses. The spectra have a complex profile, resulting from the effect of MAS on the strong ^7^*Li*–^7^*Li* dipolar coupling. The values of the average isotropic chemical shift δ_CS_ show a slight but consistent variation to higher frequencies as depicted in Figure 11a. According to data from the literature,^28^ this trend in ^7^*Li*-δ_CS_ indicates shorter *Li–O* average distances in the first coordination sphere around *Li* in glasses with more *K*.

Figure 11b displays the extension of the ^7^*Li*-*MAS*-*NMR* sideband pattern, which is proportional to the magnitude of the average quadrupolar coupling constant (*C*_q_), as a function of the substitution ratio. It can be seen that at low concentrations, *Li* ions occupy more symmetrical sites, and as the concentration increases, the local symmetry around the coordination polyhedron of *Li* decreases, resulting in an increase in the electric field gradient. The behavior of the FWHM, as shown in Figure 11c, also decreases with an increasing *K* concentration in the sample. The width of the resonance line is proportional to the homonuclear coupling strength, and as the *Li* concentration decreases, the ions tend to spread further apart, leading to a decrease in homonuclear dipolar coupling, due to the lower probability of having shorter ^7^*Li*–^7^*Li* internuclear distances.

## Discussions

The *T*_g_ values in mixed-ion glass systems exhibit a nonlinear dependency on composition, consistent with previous studies.^11,19^ This nonlinearity is typically attributed to the size difference between the two cations. The observed decrease in *T*_g_ suggests a significant weakening of the glass network as *Li*^+^ ions are replaced by *K*^+^ ions, due to the weaker Coulombic interaction between *K*^+^ and the NBOs. The intensity of the MIE for intermediate compositions in *T*_g_ is more clearly observed in Figure 1b, represented as Δ*T*_g_. However, the greatest deviation from linearity is found in the *Na–Li* system, which exhibits the smallest size difference between the cations. This suggests that cation size difference alone is not the dominant factor in determining the intensity of the MIE in *T*_g_. Instead, the degree of structural disruption caused by cation mixing plays a crucial role. In the *Li–Na* system, the relatively small difference in ionic radii (28%) leads to a more uniform distribution of cations without significant structural reorganization, resulting in a strong negative deviation in *T*_g_ due to the enhanced network mobility.

In the *K–Li* system, the significant size difference (47%) leads to structural modifications that influence both the thermal and electrical properties. The substitution of *Li*^+^ by *K*^+^ reduces the density of cross-links between phosphate chains, weakening the network and leading to the observed decrease in *T*_g_. Additionally, the larger *K*^+^ cations disrupt the homogeneous distribution of NBOs, introducing local structural inhomogeneities. This effect contributes to the nonlinear variation of *T*_g_ and is consistent with trends observed in other mixed-alkali metaphosphate systems. As the larger cations *Rb^+^* and *Cs*^+^ are introduced, their increasing size mismatch forces a partial restructuring of the phosphate network, which can mitigate the weakening effect and reduce the negative deviation. In the case of *Li–Cs*, the substantial size difference disrupts the connectivity of the phosphate chains in a way that locally stabilizes the network, leading to a positive deviation in *T*_g_.

Regarding cation–cation interactions, the increasing size mismatch between *Li*^+^ and *K*^+^ results in a greater differentiation between cationic sites, which affects ionic transport. According to the Random Ion Distribution Model (RIDM), when the cation size difference is significant, the structural adaptation to accommodate each species leads to distinct conduction pathways, increasing the energy barriers for ion migration. This effect is clearly observed in the *K–Li* system, where the mixed composition exhibits a pronounced suppression in ionic conductivity. Compared to other systems, such as *Li–Na* and *Li–Rb*, where the size mismatch is smaller, the blocking effect in *K–Li* is stronger, but not as extreme as in *Li–Cs*, where the conduction pathways become highly disrupted. These findings reinforce that the impact of cation mixing in metaphosphate glasses is governed not only by size mismatch but also by structural reorganization within the glass network.

The ionic conductivity (σ_DC_) in mixed-ion glass systems also displays a nonlinear behavior, characterized by a pronounced MIE. This effect is particularly evident at the intermediate composition (*x* = 0.5), where a minimum in σ_DC_ occurs, as shown in Figure 4. This behavior can be attributed to the substitution of a cation with a smaller atomic radius and higher ionic potential by one with a larger radius and lower potential, resulting in the obstruction of conduction pathways for both types of cations. At this composition, interference between the conduction pathways reaches its maximum, further increasing the activation energy for ionic transport.

Furthermore, as shown in Figure 5, the intensity of the MIE in σ_DC_ decreases with increasing temperature. This behavior is attributed to the high activation energies in mixed-ion systems, where the average ionic hopping rate decreases more rapidly than that in single-cation glasses at lower temperatures. The increase in the activation energy results from the partial blocking of preferred diffusion pathways, forcing ions to move along paths with higher energy barriers.

The observed MIE intensity in σ_DC_ for intermediate compositions of mixed lithium-phosphate systems (*Na–Li*, *K–Li*, *Rb–Li*, and *Cs–Li*) is directly correlated with the cation size differences, which are 28%, 47%, 50%, and 57%, respectively, relative to the largest ion. This finding aligns with a fundamental hypothesis of the RIDM,^13−18^ which postulates that ion size differences result in structurally distinct sites adapted to each ion. This structural adaptation reduces the likelihood of successful ionic jumps between sites optimized to receive different species.

The random distribution of cations in the *K–Li* system further supports the RIDM hypothesis. The nearly linear evolution of chemical shifts and FWHM in ^31^*P* and ^7^*Li* NMR data (see Figures 9 and 11) demonstrates a random distribution of *K* and *Li* cations in the glass network, excluding the possibility of phase segregation. This conclusion is corroborated by the evolution of Raman shifts (Figure 7), which exhibit clear solid-solution behavior, providing direct evidence of ionic mixing.

These findings provide important insights into the Mixed Ion Effect in phosphate glasses and the role of ionic mixing in influencing their thermal and transport properties.

## Conclusions

This study offers valuable insights into the structural mechanisms underlying the MIE in potassium–lithium metaphosphate glasses. By integrating Raman spectroscopy and NMR techniques, two fundamental hypotheses of the RIDM were validated: the structural specificity of the sites occupied by each cation species and their random distribution within the vitreous network at the atomic scale. The observed behavior of Δ*T*_g_ in the *K–Li* system, consistent with the *Na–Li* and *Rb–Li* systems, demonstrates that cation size differences alone cannot account for the intensity of the MIE. Instead, the results indicate that structural reorganization plays a crucial role, particularly in *T*_g_, as the redistribution of nonbridging oxygens and network flexibility significantly influence thermal stability. These results provide a deeper understanding of the role of ionic mixing in determining the behavior of mixed-ion glasses, underscoring the need for a more comprehensive exploration of these systems.
